# Predicting siRNA potency with random forests and support vector machines

**DOI:** 10.1186/1471-2164-11-S3-S2

**Published:** 2010-12-01

**Authors:** Liangjiang Wang, Caiyan Huang, Jack Y Yang

**Affiliations:** 1 Department of Genetics and Biochemistry, Clemson University, Clemson, SC 29634, USA; 2 School of Electrical and Computer Engineering, Purdue University, West Lafayette, Indiana 47907, USA; 3 Center for Computational Biology and Bioinformatics, Indiana University School of Medicine, Indiana University Purdue University, Indianapolis, Indiana 46202, USA; 4 Center for Research in Biological Systems, University of California at San Diego, La Jolla, California 92093-0043, USA

## Abstract

**Background:**

Short interfering RNAs (siRNAs) can be used to knockdown gene expression in functional genomics. For a target gene of interest, many siRNA molecules may be designed, whereas their efficiency of expression inhibition often varies.

**Results:**

To facilitate gene functional studies, we have developed a new machine learning method to predict siRNA potency based on random forests and support vector machines. Since there were many potential sequence features, random forests were used to select the most relevant features affecting gene expression inhibition. Support vector machine classifiers were then constructed using the selected sequence features for predicting siRNA potency. Interestingly, gene expression inhibition is significantly affected by nucleotide dimer and trimer compositions of siRNA sequence.

**Conclusions:**

The findings in this study should help design potent siRNAs for functional genomics, and might also provide further insights into the molecular mechanism of RNA interference.

## Background

RNA interference (RNAi) is a post-transcriptional gene regulatory mechanism by which a double-stranded RNA (dsRNA) induces sequence-specific gene silencing [1]. The RNAi pathway consists of multiple steps, including the cleavage of the dsRNA by Dicer to form a 19-nucleotide short interfering RNA (siRNA) with 3' overhangs, the incorporation of the siRNA molecule into the RNA-induced silencing complex (RISC), and the recognition of the target gene transcript(s) by the RISC-siRNA complex to induce mRNA degradation or translational repression. In mammals and many other organisms, chemically synthesized siRNA molecules can be introduced into cells to knockdown the expression of a specific gene. Because of the simplicity and low cost, siRNA-based gene silencing has quickly become an important technique in functional genomics [2].

Since not all siRNAs are equally effective, siRNA design is one of the critical steps in gene silencing studies. Earlier experimental data have suggested several sets of empirical rules for designing potent siRNAs. For example, Ui-Tei et al. [3] proposed several criteria for potent siRNAs, including the presence of AU-rich 5' terminal region and G/C at the 3' end of the antisense strand, and the absence of long GC stretches (>9 base pairs). The thermodynamic properties of siRNA duplexes were shown to affect target mRNA degradation [4,5]. In addition, secondary structure in siRNAs could reduce the efficacy of gene silencing [6]. Although these findings provided important insight into RNA interference, the empirical rules were often derived from relatively small datasets, and thus might not cover all the relevant features affecting siRNA potency.

With the accumulation of siRNA data, machine learning methods have been developed for both classification and regression analysis of siRNA potency. Strom [7] reported a genetic programming (GP) method with boosting algorithms for the binary classification of effective and ineffective siRNAs. It was shown that the boosted GP classifier outperformed support vector machines (SVMs) trained on the same dataset. Ladunga [8] trained SVMs for siRNA classification based on biophysical signatures of free energy, target site accessibility and dinucleotide characteristics. Wang et al. [9] also constructed an SVM classifier to identify hyperfunctional siRNAs by using simple sequence and structural features such as base composition at each position, GC content, and secondary structure. Artificial neural networks (ANNs) have been trained for regression analysis of siRNA efficacy. Huesken et al. [10] developed the BIOPREDsi model with nucleotide sequence information to predict the inhibitory activity of siRNAs, and used the ANN model to design a human siRNA library. Shabalina et al. [11] performed thermodynamic and correlation analyses on a set of siRNAs, and constructed an ANN model with three parameters characterizing siRNA sequences. In addition, Vert et al. [12] constructed the simple linear model DSIR using the LASSO procedure for siRNA efficacy prediction with basic sequence features.

The above-mentioned previous studies suggest that many siRNA features of sequence composition, thermodynamic stability and secondary structure are related to the effectiveness of gene silencing. However, few advanced methods have been used to select the most relevant features for predicting siRNA potency. Most of the previous studies selected some siRNA features based on empirical knowledge or simple statistical analysis (e.g., correlation analysis).

More recently, Klingelhoefer et al. [13] used a stochastic logistic regression-based algorithm to identify relevant features associated with siRNA potency. The feature selection method revealed several sequence motifs such as UCU in potent siRNAs.

In this study, random forests (RFs) were constructed to select important sequence features for predicting siRNA potency. RF-based variable importance measures were previously used in microarray expression data analyses to select a relatively small set of informative genes for disease/sample classification [14,15]. However, it remained to be demonstrated whether the RF methodology could be applied to sequence feature selection for siRNA classification. The selected sequence features were used to construct SVM models for predicting siRNA potency. Our results suggest that siRNA potency is significantly affected by its nucleotide dimer and trimer compositions. Some nucleotide motifs such as UCC appear to be positively correlated with siRNA efficacy, whereas other motifs such as GAG may have a negative effect on gene expression inhibition. The findings will likely be useful for rational siRNA design in large-scale functional genomics projects.

## Methods

### Data

As described in the previous study [7], a non-redundant set of experimentally evaluated siRNAs were collected from several published studies. For each siRNA, the relative level of target gene mRNA was the ratio of the remaining mRNA level after siRNA treatment to the wild-type control level. The relative mRNA level (ranging from 0 to 1) was used to determine the effectiveness of mRNA knockdown. Effective siRNAs gave rise to lower levels of remaining gene expression. The cut-off level of 0.5 was used to define positive instances (potent siRNAs with the relative mRNA level ≤ 0.5) and negative instances (non-potent siRNAs with the relative mRNA level > 0.5). The dataset used in this study consisted of 165 positive instances and 115 negative instances. Each siRNA instance was a sequence of 19 nucleotides (from 5' to 3' end) representing the antisense strand of the target gene mRNA transcript. The two-nucleotide overhang at the 3' end of a siRNA was not included in the data instance. Although more siRNA data have recently become available and will be used in the future study, our approach was first tested on this relatively small dataset so that our findings could be compared with the previously published results.

### Sequence features

Of many potential features, only a few may be relevant for siRNA classification. This study examined 120 sequence features belonging to six groups (Table 1). The first group has 19 features, each of which is the nucleotide identity (A, U, G or C) of a sequence position in a siRNA. In this study, real numeric values are used to represent the nucleotide identity: 0.1 for A, 0.2 for U, 0.3 for G, and 0.4 for C. The second feature group indicates the base composition of a siRNA (the frequency of A, U, G or C). The third group has 16 features representing the frequencies of all possible dinucleotides (e.g., AG, UC, etc). The fourth feature group consists of 64 frequencies of all possible trinucleotides (e.g., CAG, UCC, etc). The fifth feature group indicates the global and local G/C contents in a siRNA. There are 16 features, one for the overall G/C content and 15 for local G/C contents. With a sliding window size of five nucleotides, local G/C contents are calculated for all the possible windows along a 19-nucleotide siRNA sequence. The sixth group has the feature of siRNA secondary structure stability. The free energy of secondary structure was calculated by using the RNAfold program in the Vienna RNA package [16].

**Table 1:** **Potential sequence features for siRNA classification**.

Feature group	Number of features
siRNA nucleotide sequence	19
Single-nucleotide frequencies	4
Dinucleotide frequencies	16
Trinucleotide frequencies	64
Global and local G/C contents	16
Secondary structure stability	1

### Random forests for feature selection

In this study, important sequence features for siRNA classification were identified by using the random forest (RF) algorithm implemented in the software package available at http://www.stat.berkeley.edu/~breiman/RandomForests/. The RF algorithm uses a combination of independent decision trees to model data and measure variable importance [17]. Each decision tree in a forest is constructed using a bootstrap sample from the data, and about one-third of the data instances are not used to grow the tree. These instances are called the out-of-bag (oob) data for the tree. At each node of the tree, *m* variables out of all the *n* input variables *(m « n)* are randomly selected, and the tree node is split using the selected *m* variables. The random selection of features at each node decreases the correlation between the trees in the forest. Thus, the RF algorithm can handle many redundant features and avoid model overfitting. It has been shown that RFs outperform AdaBoost ensembles on noisy datasets, and can perform well on data with many weak input variables [17].

To evaluate the importance of variable *x*, its values in the oob instances associated with each tree in the forest are permuted randomly. The permuted oob instances as well as the original oob instances are then classified using the tree. The number of correct classifications on the original oob instances is subtracted by the number of predictions for the correct class on the permuted oob instances to calculate a raw score based on the tree. The importance score of variable *x* is defined as the average of raw scores over all the trees in the forest. For a fixed number of trees in the forest, the larger importance score a variable has, the more important it is for classification. In addition, a z-score can be obtained by dividing the variable importance score by its standard error, and a statistical significance level may be assigned to the z-score assuming normality [17].

### Support vector machine classifiers

Support vector machines (SVMs) were trained with the selected sequence features to predict siRNA potency. The *SVMlight* software package (available at http://svmlight.joachims.org/) was used to construct SVM classifiers. The SVM learning algorithm has been applied to a variety of biological problems for pattern classification, and may have superior generalization power with the ability to avoid overfitting [18]. For a given set of binary-labelled training examples, the SVM algorithm maps the input space into a higher-dimensional space, and seeks a hyperplane to separate the positive data instances from the negative ones [19]. The optimal hyperplane maximizes the separation margin between the two classes of training data, and is defined by a fraction of the input data instances (called support vectors) close to the hyperplane. The distance measurement between the data points in the high-dimensional space is defined by the kernel function. In this study, we used the radial basis function (RBF) kernel:(1)

where and are two data vectors, and γ is a training parameter. A smaller *γ* value makes the decision boundary smoother. Another parameter for SVM training is the regularization factor C, which controls the trade-off between low training error and large margin [19]. Different values for the *γ* and *C* parameters have been tested in this study to optimize the classifier performance.

### Classifier performance evaluation

We used a fivefold cross-validation approach to evaluate the performance of SVM classifiers.Positive and negative instances were distributed randomly into five folds. In each of the five iterative steps, four of the five folds were used to train a classifier, and then the classifier was evaluated using the holdout fold (test data). The predictions made for the test instances in all the five iterations were combined and used to compute the following performance measures:(2)(3)(4)(5)

where *TP* is the number of true positives; *TN* is the number of true negatives; *FP* is the number of false positives; and FN is the number of false negatives. In addition to overall accuracy, sensitivity and specificity, Matthews correlation coefficient (MCC) is also commonly used as a measure of the quality of binary classifications [20]. MCC measures the correlation between predictions and the actual class labels.

The Receiver Operating Characteristic (ROC) curve is probably the most robust approach for classifier evaluation and comparison [21]. The ROC curve is drawn by plotting the true positive rate *(i.e.,* sensitivity) against the false positive rate, which equals to (1 – specificity). In this work, the ROC curve has been generated by varying the output threshold of a classifier and plotting the true positive rate against false positive rate for each threshold value. The area under the ROC curve (AUC) can be used as a reliable measure of classifier performance [22]. Since the ROC plot is a unit square, the maximum value of AUC is 1, which is achieved by a perfect classifier. Weak classifiers have AUC values close to 0.5.

## Results and discussion

### Random forest-based selection of important features

There are many potential features for siRNA classification. To select the important features, siRNA sequences were coded with the 120 features shown in Table 1, and then used to construct random forests (RFs) with different settings of the *m* parameter (the number of variables randomly selected to split each node in a tree). As suggested by the RF software [17], the ceiling of the square root of the total number of input variables might be used as the default value of *m* (i.e., *m =* 11). In this study, 10 RFs were constructed by varying the *m* parameter setting from 2 to 20 *(m =* 2, 3, 5, 7, 9, 11, 13, 15, 17, or 20). Each RF with 1000 trees selected the top 20 features based on the z-score of variable importance. Some of the common features selected by the RFs were then identified for siRNA classification. The use of multiple RFs might increase the reliability for identifying relevant features.

Table 2 shows the important features that were selected by at least 5 out of the 10 RFs. The average values of raw scores and z-scores of variable importance are shown together with the feature's correlation with siRNA efficacy (inhibition of target gene expression). Interestingly, the efficacy of gene silencing appears to be significantly affected by nucleotide dimer and trimer compositions of siRNA sequence. For instance, the most important feature is the frequency of UCC on the antisense strand (UCC% in Table 2). This feature was selected by all the 10 RFs with an average z-score of 14.909 (statistical significance level *p* = 0). Moreover, the frequency of UCC was found to be positively correlated with siRNA efficacy (Pearson's correlation coefficient *r =* 0.294). The frequency of UC was also selected as an important feature by the RFs (z-score = 11.255, and *r =* 0.281). The other composition features positively correlated with siRNA efficacy include CG%, AAG%, AUC%, GCG%, AAC%, UUU%, ACA%, UUC%, and CAA% (Table 2).

**Table 2:** Important features selected by random forests.

Feature	#RFs	Raw score	Z-score	Correlation
UCC%	10	2.461	14.909	0.294
CAG%	10	1.514	11.849	-0.289
GAG%	10	1.652	11.674	-0.305
UC%	10	1.988	11.255	0.281
GCA%	10	1.140	11.191	-0.265
G%	10	1.672	9.483	-0.266
CG%	10	1.235	8.460	0.133
AUA%	10	0.524	8.148	-0.166
AAG%	9	0.848	7.851	0.102
CUG%	10	0.918	7.240	-0.173
U%	9	1.201	7.170	0.127
G/C% (first 5 bases)	10	1.075	7.116	-0.256
AUC%	8	0.632	6.565	0.201
AG%	8	0.910	6.557	-0.277
GG%	9	0.831	6.478	-0.190
GCG%	6	0.554	6.422	0.059
G/C% (overall)	5	0.959	6.414	-0.147
GGA%	7	0.717	6.326	-0.218
AAC%	10	0.409	6.326	0.162
UUU%	9	0.714	6.317	0.108
GGC%	9	0.595	6.304	-0.134
NT3 (C)	5	0.901	6.258	-0.199
ACA%	8	0.473	6.218	0.092
UUC%	7	0.542	5.897	0.125
CC%	7	0.704	5.807	0.004
CAA%	6	0.432	5.602	0.129

Some trinucleotide or dinucleotide features show negative correlation with siRNA efficacy. For example, the frequencies of CAG and GAG on the antisense strand have average z-scores of 11.849 and 11.674, respectively, and are negatively correlated with siRNA efficacy (Table 2). The Pearson's correlation coefficients are -0.289 for CAG% and -0.305 for GAG%. The other composition features with negative effects on siRNA efficacy include GCA%, AUA%, CUG%, AG%, GG%, GGA%, and GGC% (Table 2). These nucleotide motifs should be avoided in designing potent siRNAs.

Several features of base composition and G/C content were also selected by the RFs. As shown in Table 2, the frequency of G on the antisense strand (G%) is negatively correlated with siRNA efficacy (*r* = -0.266), whereas U% shows a positive correlation with gene expression inhibition (*r* = 0.127). Both the global G/C content and the G/C content in the first 5 bases (antisense strand) are negatively correlated with siRNA efficacy (*r* = -0.147 and -0.256, respectively). In addition, the nucleotide identity at the third position (NT3, from the 5' end) of the antisense strand may be an important feature for siRNA classification. A nucleotide C at the third position has a negative effect on siRNA efficacy *(r =* -0.199). However, the nucleotide identities at the other positions as well as siRNA secondary structure stability were not selected by most of the RFs.

Some of the important features in Table 2 were previously shown to be related to the efficacy of gene silencing. Very high G/C contents were found to have a negative effect on siRNA efficacy [9,13]. The presence of AU-rich 5' terminal region of the antisense strand was proposed to be one of the criteria for designing potent siRNAs [3]. Consistent with the previous findings, both the overall G/C% and the 5' terminal G/C% (first 5 bases) of the antisense strand were selected by the RFs in this study. It was previously shown that the frequency of U, but not G or GG, was positively correlated with siRNA efficacy [8]. This observation has also been confirmed in the present study. Nevertheless, many of the other features in Table 2, including the top three features (UCC%, CAG%, and GAG%), have not been well documented in the literature. Thus, the findings in this study provide new insights into the rational design of potent siRNAs.

### Support vector machine classifiers of siRNA potency

To validate the important features selected by the RFs, support vector machines (SVMs) were trained with these features for siRNA classification. The siRNA instances were labeled with either 'potent' (positive instances showing ≥50% reduction in gene expression) or 'non-potent' (negative instances showing <50% reduction in gene expression). Classifier performance was evaluated using a fivefold cross-validation approach. Different settings of SVM training parameters were tested, and the best classifier was constructed with *γ =* 1.2 and *C =* 1.2. As shown in Table 3, the SVM classifier, named RF_Features, achieved 70.71% overall accuracy with 73.94% sensitivity and 66.08% specificity. The Matthews correlation coefficient (MCC) was 0.3983, and the area under the ROC curve (AUC) reached 0.7529. Thus, the features selected by the RFs can be used to construct relatively accurate SVM models for predicting siRNA potency.

**Table 3:** Performance of support vector machine classifiers.

Classifier	Accuracy (%)	Sensitivity (%)	Specificity (%)	MCC	AUC
RF_Features	70.71	73.94	66.09	0.3983	0.7529
All_Features	68.93	76.97	57.39	0.3499	0.7372
Seq_Features	65.36	68.48	60.87	0.2912	0.6624

**Figure 1:**
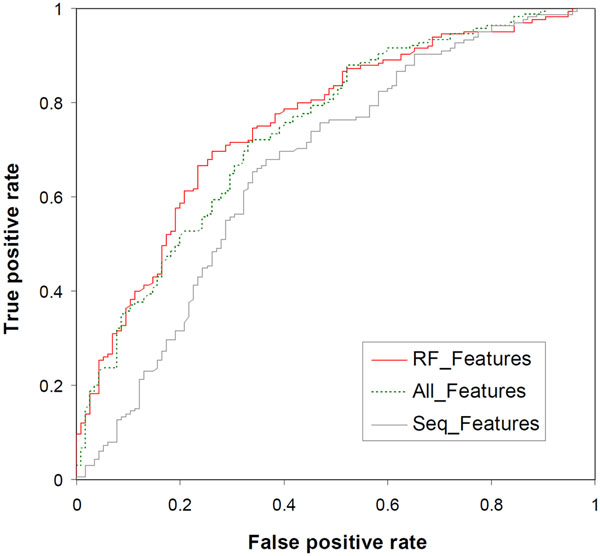
Classifier performance evaluation using ROC curves.

For performance comparison, SVM classifiers were also constructed using all the 120 features or only the 19 siRNA sequence features (Table 1). As shown in Table 3, the classifier RF_Features outperformed these two classifiers, namely All_Features and Seq_Features. The All_Features classifier (constructed with all the 120 features) achieved 68.93% overall accuracy with 76.97% sensitivity and 57.39% specificity, MCC = 0.3499, and AUC = 0.7372. The results suggest that there may be some redundant or correlated information in the full feature set, and the RF-based feature selection can be used to improve classifier performance. The Seq_Features classifier (constructed using the 19 siRNA sequence features) showed significantly worse performance than RF_Features (Table 3), suggesting that the selected composition features contain some important information for siRNA classification. In Figure 1, the Receiver Operating Characteristic (ROC) curves of the three SVM classifiers are compared, and the result is consistent with the performance measures shown in Table 3. The classifier RF_Features appears to be slightly more accurate than All_Features. Both RF_Features and All_Features are significantly better than Seq_Features.

**Figure 2:**
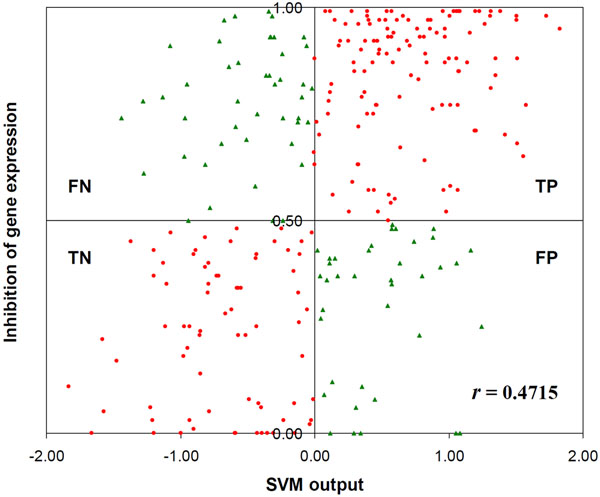
**Correlation of SVM output with siRNA efficacy.** The true positive (TP) and true negative (TN) predictions are shown in red circles, whereas the false positive (FP) and false negative (FN) predictions are shown in green triangles.

To further evaluate the classifier RF_Features, we examined the SVM output used to predict siRNA potency. The classifier was constructed using siRNA instances with binary labels (potent or non-potent). The actual magnitude of gene expression inhibition was not included in the training data. In Figure 2, the SVM output for each test instance is plotted against the level of gene expression inhibition. Interestingly, the output from RF_Features is positively correlated with the level of gene expression inhibition (Pearson's correlation coefficient *r =* 0.4715). The result further suggests that the classifier RF_Features has learned some important siRNA patterns related to the efficacy of gene silencing.

The classifier RF_Features compared favorably with several existing models. In the previous study by S trom [7], a similar dataset of siRNAs was used to construct SVM and genetic programming (GP) classifiers. The most accurate SVM classifier achieved an AUC of 0.70, MCC = 0.31, 61% sensitivity and 68% specificity. The boosted GP classifier showed better performance with AUC = 0.72, MCC = 0.24, 50% sensitivity and 73% specificity. In this study, the classifier RF_Features achieved an AUC of 0.7529, MCC = 0.3983, 73.94% sensitivity and 66.09% specificity. However, it is not straightforward to compare RF_Features with the other existing models. In two previous studies [8,9], SVMs were also trained for siRNA classification, but with different definitions of positive and negative instances. In several other studies [10-12], regression models instead of classifiers were constructed for predicting siRNA efficacy.

## Conclusions

We have developed a new machine learning approach for predicting siRNA potency based on random forests and support vector machines. Since there were many potential features for siRNA classification, random forests were used for feature selection based on variable importance scores. Interestingly, most of the selected features were nucleotide dimer and trimer compositions of siRNA sequence. Some nucleotide motifs (e.g., UCC) showed positive correlation with siRNA efficacy, whereas other motifs (e.g., GAG) might have a negative effect on gene silencing. These important features were used to train support vector machines for predicting siRNA potency with relatively high accuracy. In the future, we will apply our approach to a large, integrated dataset of siRNAs, and develop a software system for rational siRNA design in functional geneomic studies.

## Competing interests

The authors declare that they have no competing interests.

## Authors' contributions

LW initiated and designed the study. LW and CH conducted the data analysis. LW drafted the manuscript. JYY participated in experiment design, result interpretation and manuscript preparation.
